# “An Active, Productive Life”: Narratives of, and Through, Participation in Public and Patient Involvement in Health Research

**DOI:** 10.1177/1049732320961053

**Published:** 2020-10-03

**Authors:** Joanna Reynolds, Ruth Beresford

**Affiliations:** 1 Sheffield Hallam University, Sheffield, United Kingdom

**Keywords:** participation, narrative interviews, PPI, involvement, health research, public, qualitative, United Kingdom

## Abstract

Public and patient involvement (PPI)—engaging the public in designing and delivering research—is increasingly expected in health research, reflecting recognition of the value of “lay” knowledge of illness and/or caring for informing research. Despite increased understanding of PPI experiences within the research process, little attention has been paid to the meaning of PPI in other areas of contributors’ lives, and its value as a broader social practice. We conducted repeated narrative interviews with five experienced PPI contributors from the United Kingdom to explore how meaning is constructed through narratives of PPI in relation to their broader “life-worlds.” Narratives were extremely varied, constructing identities and meanings around PPI in relation to family and social life, career and employment, financial status, and wider social agendas, as well as health. This emphasizes the importance of recognizing PPI as a social practice with diverse meaning and value beyond health research.

## Introduction

The involvement of patients and the public in the design and delivery of health research has become an established and expected practice in the United Kingdom and internationally. Typically termed “public and patient involvement” (PPI), it reflects a range of moral, democratic, and instrumental arguments around valuing and representing the “voice” of the potential beneficiaries of health research. It can also be situated in a broader “participatory shift” (Madden & Speed, 2017), characterized by increasing opportunity and expectation for citizen contribution to policy and decision-making processes. PPI can be considered a valuable mechanism to help address the democratic deficit within traditional research structures (Schwartz et al., 2019; Thompson et al., 2012). Yet, there are continuing critiques of whether PPI can actually transform the structural relations of research and/or have positive impact on health outcomes (Green, 2016). Attention has been paid to the status and experience of the PPI contributor within the research setting and its hierarchies of knowledge (Locock et al., 2017). However, what has received far less attention is how the role of the PPI contributor intersects and is negotiated in other areas of people’s lives, not just within the health research context. To consider how PPI might be understood as a meaningful social practice, this article describes how experienced PPI contributors situate their PPI experiences in broader narratives of their lives.

### Mapping the PPI Landscape

The “imperative” (Madden & Speed, 2017) for PPI in health research has evolved over the past couple of decades, preceded by established practices of involvement in health policy and health services planning and delivery, and in the charity and voluntary sectors (Green, 2016; Russell et al., 2019). In the United Kingdom, the PPI landscape has been dominated recently by the “architecture” of health research funding and governance (Thompson et al., 2014). Large funding bodies, such as the National Institute for Health Research and Medical Research Council, now expect public involvement across multiple stages of the research process (Hughes & Duffy, 2018). The U.K. public involvement advisory group, INVOLVE, has been influential in shaping definitions of and expectations for PPI in research (Rose, 2014), for example, producing guidance recommending financial recompense for PPI contributions, in addition to payment of expenses (INVOLVE, 2020).

Concerns over public involvement in health research as a “tokenistic” practice (Brett et al., 2014; Madden & Speed, 2017) have led to more calls for evaluating its effects (Crocker et al., 2017). These evaluations can be framed in consumerist or rationalist terms (Beresford, 2003), for example, assessing how PPI impacts on the efficiency of the research process through improving participant recruitment, but increasing costs (Brett et al., 2014). From a more democratic and moral framing, PPI could be evaluated in terms of its capacity to transform the established hierarchies of power and knowledge: the “social relations of research” (Green, 2016, p. 10). Further research has focused on the impact of PPI on the contributors themselves, highlighting positive effects of being involved on contributors’ skills, knowledge, and friendships (Staley, 2009), and on self-identity, perceived capacity (Thompson et al., 2014), and emotional well-being in relation to their health status (Werner-Seidler & Shaw, 2019).

Yet, the framing of this literature remains predominantly research-centric: interpreting PPI and its impact within the context of the research process, rather than in the context of the contributors’ broader lives. Sociologists have applied Habermas’s theory of communicative action (Habermas, 1989) to understanding patient and public contribution to research and other decision-making (Bissell et al., 2018; Williams & Popay, 2001). Following their work, exploring the meaning of PPI only in relation to health research could reflect Habermas’s description of the “systematization” of the “life-world.” This is the encroaching of the modes of material reproduction of society—here, the health research knowledge production “system”—into the sphere in which knowledge and understanding are culturally produced: the “life-world” of PPI contributors’ everyday lives. To understand more deeply the value and impacts of PPI as a social practice, and its impacts, it is important to examine PPI’s meaning from the starting point of contributors “life-worlds,” rather than the starting point of the “system” (the health research context).

### Conceptualizing the “Lay,” Expert

PPI literature has explored identity(ies) of the PPI contributor, particularly in relation to their “lay” status within the research context, and the level of representativeness of contributors in relation to the broader patient and public population. Critical debates have highlighted the tensions in the PPI contributor as both “expert” by nature of their lived experience of a health condition, service, or caring role, and as “lay” or nonexpert by nature of not being a professional in the research field (Maguire & Britten, 2018; Martin, 2008; Thompson et al., 2012). Debates around the perceived “professionalization” of PPI contributors who build up new skills, knowledge, and capital of the research process itself through multiple PPI roles complicate this further. PPI contributors may be considered at risk of losing their valuable “grassroots credibility” (Thompson et al., 2012, p. 612), and their capacity, from a “naïve” position, to question the norms of the research process. Yet, to be able to participate meaningfully in formal research spaces, PPI contributors may need to acquire and demonstrate particular forms of capital (Locock et al., 2017) that go beyond the symbolic capital of their lived experience.

Discursive shifts toward “co-production” of research between researchers and patients/public offer some countenance to these tensions, highlighting the need for user-led research as a mechanism for inverting the traditional power structures of the research world (Hughes & Duffy, 2018). However, this literature continues to frame PPI contributors’ identities, status and capital in relation to the health research context, or “system,” only, for example, examining contributors’ relationships with researchers (Locock et al., 2017), identity within research spaces (Maguire & Britten, 2018), and impact on research design (Thompson et al., 2014). This means that the “value” of PPI in relation to other spheres of meaning outside health research, with potentially more relevance to the contributors themselves, is overlooked. To develop further our critical understanding of PPI as a practice of social value, and to move beyond these problematic framings of “lay” and “expert,” is important for us to see how the meaning of PPI is constructed beyond the health research system, in relation to other dimensions of contributors’ lives, or “life-worlds.” Our research sought to answer the questions: How do PPI contributors situate their experiences of public involvement in the context of their broader lives, over time, and how are meaning and identities constructed through narratives of these experiences?

## Method

We undertook a qualitative approach involving repeat, in-depth interviews with experienced PPI contributors from the United Kingdom to elicit narratives of their experiences of PPI in relation to other areas of their lives.

### Theorizing Narratives

Our methodological approach draws on the rich body of sociological work examining narrative construction of identity. Following Galvin (2005), we see narratives as a form of storytelling which acts both as a process of continuous construction of identity(ies) and as a form of agency in their telling and shaping of the individual’s own reality. As Hurdley (2006) argues, “the analysis of personal, narrative accounts is a powerful means to understand the construction and performance of selves” and is valuable for considering “transformations in identity” (p. 719), and the temporal character of experience and self (Franzosi, 1998). We acknowledge narratives as socially situated accounts that emerge through interaction and negotiation, offering both stories of experience and scripts for positioning the self (Rhodes et al., 2010).

In medical sociology, narrative approaches have been widely employed for understanding experiences of illness (Thomas, 2010) and treatment (Vindrola-Padros & Johnson, 2014), and how identities are constructed through narration of ongoing relationships between the “body, self and surrounding world” (Hydén, 1997, p. 51). Narratives have also proved valuable for exploring the representation of experiences and identities among marginalized groups such as care-leavers (Evans, 2019). This reflects something of the origins of narratives in medical sociology, as a mode of giving voice to experiences that lie outside the dominant structures of biomedicine (Hydén, 1997). As such, narratives offer valuable scope for extending the work on PPI, facilitating constructions of meaning around PPI experience in relation to the “surrounding worlds” of participants, and beyond the dominant framing of health research.

### Sample and Recruitment

We adopted a blend of convenience and purposive sampling to recruit five people from the United Kingdom with considerable experience of doing PPI work in the context of health research. We defined “experience” as having contributed to three or more health research studies in a PPI capacity in the past 10 years, and deliberately defined “health research” broadly, to include clinical, health services, public health, and other related research. To identify potential participants, we shared information and an invitation to participate round existing PPI networks in the United Kingdom, via researchers and other contacts working in the PPI field, and on social media, and asked those interested to contact us with a brief summary of their PPI experience.

We were contacted by many (more than 30) PPI contributors who fulfilled our basic inclusion criteria, and several sent detailed “CVs” of their PPI experience. We then selected participants purposively from those interested to capture a range of types of health research experience, and demographic identities, specifically gender and age. We deliberately chose a small sample of five, reflecting our aim to explore in depth and over multiple occasions how individuals positioned themselves in relation to PPI through extended narratives. We wished to privilege individualized experiences and identities, rather than seeking to draw broader, more generalized interpretations from across the sample of participants.

### Interviews

We conducted two in-depth interviews with each participant, with between 4 and 6 weeks between each interview. Repeat, or serial, interviews have been identified as a valuable approach for building deeper and more trusting interactions between participants and researchers than possible in a single encounter (Read, 2018). Furthermore, we felt that one interview would not offer sufficient time for participants to consider their PPI experiences in relation to multiple different areas of their lives (e.g., family, employment, health, social life, and others). Instead, we thought two interviews would enable a fuller understanding of the narrative constructions of identity and meaning in relation to these experiences, and to understand better how “different versions of narratives are related” in response to the social context of the research encounter (Hurdley, 2006, p. 730).

Participants chose to be interviewed either in their home or in a meeting room at the university and were interviewed by Joanna Reynolds or Ruth Beresford. For each participant, both the first and follow-up interviews were conducted by the same researcher. In the first interviews, participants were encouraged to “tell their story” and this was supported by a loosely structured set of four broad questions: (a) how they got involved with health research and what else was happening at the time, (b) experiences of doing PPI work, (c) how PPI work has intersected other areas of life, and (d) how they view the different roles they play/have played. The second interviews were informed by reflections from the first and were used to follow up on specific aspects of participants’ experiences, clarify any details, and allow participants the opportunity to go into more depth around issues they considered important. First interviews lasted between 57 and 117 minutes (*M* = 85 minutes) and second interviews lasted between 48 and 137 minutes (*M* = 87 minutes). All interviews were audio-recorded with participants’ permission and transcribed verbatim.

### Analysis

While much literature on analyzing narrative interviews has followed a structuralist approach, identifying formal components of narratives (Jovchelovitch & Bauer, 2000), we took an approach that reflected more the “holistic” perspective recommended by Earthy and Cronin (2008), which seeks to explore meaning and change of narrated events within the context of the participant’s life. We did this in three stages, focusing on each participant in turn, led by Joanna Reynolds. First, the researcher read closely the transcripts and reflections from each interview, to draw out and map by hand a chronological story for each participant, relating to PPI and their broader lives, structured loosely as “starting point,” “progression,” “current position,” and “future.” Second, the story was interpreted to elicit which personal identities were being presented and how, and the meaning attached to these through their narration. Third, the story was further interpreted to identify how the participant situated themselves and their experiences in relation to broader social and political stories, what Evans (2019) describes as “master narratives”: “cultural storylines” that constitute shared understanding between narrator and listener (p. 32).

### Positionality of the Researchers

As researchers we both identified with an interpretivist-constructivist philosophical position in this study. Joanna Reynolds’s interest in the topic emerged through experience of coordinating PPI for previous public health research and building up relationships with PPI contributors in this role. Ruth Beresford did not have direct experience of PPI in health research but has research experience in the methods and principles of co-production. We both view PPI as a practice of sociological interest. Discussion before and after each interview, and throughout analysis, helped us to explore and account for positionalities that might have shaped interactions with participants. Those of particular note, given the focus of the interviews, included age (we were between 10 and 35 years younger than each participant), educational and professional status (we both have doctorates and are employees of an academic institution), and current health status (we both identify as able-bodied with no serious health conditions).

### Ethical Considerations

This study was approved by the ethics committee of Sheffield Hallam University, reference number ER12959967. Those people selected for participation were sent copies of the participant information sheet and invited to indicate if they were still happy to participate (none declined). Prior to the first interview, participants were given a copy of the consent form and the opportunity to ask questions, before giving written consent. They were reminded that while we hoped they would participate in two interviews, they had the right to withdraw from participation at any point, and they were asked to sign a second consent form prior to the second interview. Participants received a £25 shopping voucher for each interview.

Acknowledging the potential for sensitive and personal experiences to emerge in the interviews, anonymity of names, organizations, and other identifying details was assured to the participants, and they were invited to choose a pseudonym for the reporting of the research. Participants were also asked to review a draft of the write-up of their narrative to check they were happy with the level of anonymization.

## Findings

Below we present in detail key elements of the narratives of each participant. For each, we first describe the personal trajectories and identities constructed through the narrative of their PPI experiences, and second, we highlight the broader narratives represented and engaged within the participant’s account. Table 1 presents the key characteristics of each participant.

**Table 1.** Summary of Participant Characteristics.

Participant	Approximate age	Number of years’ experience of PPI
Kendra	60s	16
Bhai	40s	7
Kat	60s	16
Brendan	50s	2
Grace	50s	20

## Kendra: “Developing Something Quite Special”

### Personal Trajectory and Identity

The diagnosis of a chronic condition that “just came out of the blue” was Kendra’s starting point for narrating her PPI journey. She described this coinciding with a period in her life when she was caring for a family member with a terminal illness. Following the death of the family member, she recalled looking for positive outcomes from negative experiences, such as fundraising for a cancer charity which led to a memorable visit to the charity’s research laboratories: “I knew I wanted to be part of it.”

Kendra characterized her involvement in PPI work from this point as a process of upward progression, first joining a local health forum and undertaking a range of projects, and taking up regional and national PPI opportunities, including sitting on a national guideline development group. Her narrative was characterized strongly by a sense of “moving forwards” in PPI work. She recounted “working [her] way through the ranks” of PPI, and pursuing opportunities with increasing responsibility and status, such as applying for “co-applicancy” on a research project. She conveyed excitement in recounting the increasing successes she had achieved in PPI, and her imagined future in the field, which she felt could lead to something “quite special.” This was set in contrast to her previous professional life working in local government, which she depicted as having been rather frustrating and unfulfilling:

*if I’m honest about not having achieved what I wanted to in my thirty-five years’ working life . . . I was never in the right place at the right time . . . and it never happened. I got certain qualifications but it didn’t seem to lead me anywhere.*

While Kendra’s narrative constructed her as increasingly expert in PPI, on a pathway toward becoming “a researcher in [her] own right,” she also expressed concern that this may lead to “losing the experiential side” of her personal health and caring experiences. Across the two interviews, Kendra revealed a range of mental and physical health experiences, and caring, family, and housing experiences, that had shaped her choices of PPI work and the knowledge she felt she could contribute. Interestingly, the health condition that was framed as the starting point for her involvement in PPI was also largely absent from Kendra’s narrative, and she acknowledged that it was typically pushed to the background of her life, and through PPI work, she was leading an “active, productive life.” She emphasized clearly how much she prioritizes PPI work over other aspects of life: “I would never sacrifice the PPI to have a better social life, to have more holidays.”

While Kendra lives alone with few family members nearby, her accounts portrayed dynamic social relations around her PPI work. She talked of navigating the emotional dimensions of relationships with researchers with whom she developed long-standing connections over time, for example, occasionally feeling “abandoned” when researchers moved on to other roles. She identified that PPI is an “unknown area” to many of her existing friends, and the amount of time she dedicates to PPI work meant she was losing touch with some of them. However, Kendra also described creating space for new social connections and friendships through PPI, for example, with other contributors. She also portrayed her ongoing PPI work as an emotional safeguard, enabling her to consider taking more “risks” in seeking personal relationships, something she had not felt comfortable doing for a long time.

### Broader Narratives Represented

Kendra’s account of PPI providing meaningful trajectory of work and increasing status also engaged broader narratives relating to the role of money in relation to PPI. Kendra drew on the narrative of the “usual suspects” of PPI work, distinguishing herself from the retired, middle-class, and professional people who are often thought to be overrepresented in PPI (Eccles et al., 2018). She presented herself as “not very well off” or “very well educated,” thus defending the importance to her of the financial recompense from PPI work, which she framed as building up her own “little business.” However, Kendra also aligned herself with the motivation of altruism for doing PPI work. When talking about the financial aspects, she drew on the familiar narrative of wanting to “give something back,” and described taking on some PPI roles in a voluntary capacity to indicate that her “heart’s in the right place.”

## Bhai: “None of This Was Planned”

### Personal Trajectory and Identity

Bhai situated the beginning of his PPI experience in his caring responsibility for a close family member. He described being invited to join a patient and carer forum at a local mental health trust after expressing frustrations around accessing appropriate care for his family member. He framed his original motivations as being keen to improve care for his family member and other patients, but then described a trajectory of taking on an increasing range of PPI opportunities across multiple research and other health service fields. Similar to Kendra, Bhai’s narrative conveyed a sense of progression as the opportunities “mushroomed” and he was offered “bigger roles,” including on national-level committees. He described learning early on about navigating the PPI “system,” “building up that portfolio of work at local level” which then enabled him to be “competitive” in his applications for national-level opportunities, such as a research funding panel.

Bhai’s narrative incorporated detailed descriptions of the wide range of PPI roles he had undertaken. These reflected multiple aspects of his identity as a carer, which he presented as enabling him to contribute valuable knowledge from his “lived experience” across different parts of the health system. He also articulated his identity as a “tax-payer” and “citizen” as validation of his right to contribute to other kinds work in an involvement capacity, such as medical education. Bhai highlighted a number of “successes” where he was able to identify the outcomes of his contributions, such as assisting a researcher with a funding application: “I’m an expert so overhauled her grant application . . . bingo!.”

However, a strong characteristic of Bhai’s account was that of a disrupted personal narrative. He framed this in relation to a cultural expectation for the traditional pathway of education, job, and marriage and described how the need to care for family members had challenged his ability to follow this pathway: “I would have been married, I would have a mortgage.” Instead, for Bhai PPI had become a “substitute for real work,” both in terms of financial recompense and meaningful activity. His narrative constructed his current situation as frustrating and unfair given his aspiration and expectation for a more formal career:

*The idea was A levels, get a degree. My undergraduate degree got interrupted because of [family member]’s health and I didn’t do as well as I could so then I had to take a number of rubbish jobs to put money in the bank to go back and do two Masters. . . . and the idea was to get a full time job at Masters level. That didn’t materialize because I’d had long periods of unemployment, being on the sick as a carer for [family members].*

This perspective intersected Bhai’s detailed descriptions of the financial dimensions of doing PPI work, including scheduling different PPI opportunities to maximize the pay offered, and navigating the different bureaucratic finance systems of the universities and organizations with whom he worked to claim back pay and expenses. This contributed to a sense of shame conveyed in Bhai’s narrative about the inferiority of PPI work in absence of “real” employment: “I’m begging for work . . . it’s piecemeal and it’s embarrassing.”

### Broader Narratives Represented

Similarly to Kendra, Bhai presented himself in opposition to the “usual suspects” narrative of PPI contributors in being younger and not receiving “state pensions and private pensions.” This highlighted the necessity of (paid) PPI opportunities for “helping [him] survive on a weekly basis.” The broader narrative of the authority of the PPI perspective was also present in Bhai’s accounts, in occasionally conflicting ways. Bhai emphasized the importance of his own capacity to be a “strong . . . advocate” through PPI work for his family members and others receiving care, and acknowledging that not everyone has a voice in this way. However, he also narrated experiences of a lack of status, particularly in the context of high-level, national committees where he felt, as a PPI contributor, he was considered a “second-class citizen” and lacked the “credentials” of senior clinicians to have influence in these spaces.

## Kat: “Go Under the Radar and Get Something Done”

### Personal Trajectory and Identity

Similarly to Kendra, Kat’s PPI engagement commenced following a serious health event, a cancer diagnosis: “the whole world sort of falls apart.” She described exploring options for managing her health while undergoing treatment, framing it as “the patient’s duty,” despite being told by a doctor that she “couldn’t do anything for [herself].” This desire for some control over her situation led Kat to a cancer and palliative care conference where she learned of opportunities to undertake PPI. She also recounted becoming motivated to get involved by the “callousness” displayed by some clinicians, and their patronizing attitude toward patients: “[they] clearly thought we knew nowt [nothing].” At these early stages, Kat described being “very skillfully guided” through the PPI process by supportive researchers, but also experienced antipathy toward patient perspectives, including, on one occasion, a sexist comment on her “limited” value to the research process.

Kat’s narrative of becoming increasingly involved in PPI opportunities conveyed a sense of the “opening up” of the research process, and she described pursuing a range of PPI roles across the cancer field, choosing “very carefully” to enable her to understand a wider picture of health research. These included roles on a cancer research panel, an ethics committee and a funding panel for health research, and she depicted a sense of personal progression to becoming an experienced PPI contributor. Describing more recent choices, Kat’s narrative depicted a shift toward using PPI roles to explore her own personal areas of research interest including nutrition and her “current little soapbox,” use of patient data. Reflecting on this range of experience she suggested she might have a more “rounded perspective” of the whole scientific process than others, including researchers, who are confined to one stage only.

Increasing involvement with PPI intersected Kat taking early retirement from a stressful job in the science and technology field, and Kat conveyed the PPI work as a valuable pastime to keep herself occupied, constructing herself as someone with “an enquiring mind.” Her narrative indicated changing family and social relationships around PPI also. Kat talked of feeling concerned that her husband might feel “left out” as she got increasingly busy with PPI work, but found some small ways for him to be involved such as helping her prepare presentations. Stating that he had been “devastated” by her diagnosis, she was relieved that assisting her PPI work helped to take “his mind off all that.” Kat also reflected that PPI roles offered more opportunities to be sociable with different people, something she admitted she sometimes finds challenging. Kat presented herself as a “bit of a geeky person” who “doesn’t socialize easily,” but identified that she had developed more self-confidence through PPI, which contrasts with the “solitary” hobbies she was more used to such as reading.

The narrative of the development of Kat’s PPI experience over time contrasted with the limited deterioration of her health, such that she identified herself as unusual in being “still around after 16 years.” She connected with a collective identity of “cancer patient” when describing an impatience for research, and a desire to “push the envelope,” given the perceived uncertainty of her future. Reflecting on her recent work encouraging open access to research data, Kat identified that she, among other experienced patients, held “soft power” through which change can be effectively pushed for but without the related risks faced by professional researchers. She portrayed herself as increasingly demanding and “more adventurous” in terms of her expectations of research and science. However, she also recognized frustrations and limitations in her role and capacity for influence through PPI, for example, not being able to shape research questions: “one thing I’ve never managed to crack is [successfully] suggesting a topic for research.”

### Broader Narratives Represented

Kat’s account also engaged with the familiar PPI altruistic narrative of “giving back,” but she also positioned herself in opposition to this, often casting her involvement in PPI as “fairly selfish” because it satisfies her personal “curiosity” and helps to keep “the mind going.” However, other parts of her narrative conveyed her passion for addressing poor experiences of care and “little injustices” faced by others:

*I was collecting information for this talk and somebody came back to me. She’d had neck cancer. And because she didn’t have advice on how to eat it’s left her with a permanently disabling condition that affects her for the rest of her life, and it’s just because it wasn’t done in time . . . I’m keen on pushing the patient point of view because that sort of thing needs to be looked at. The next patient might present with the same problem.*

Kat’s account also reflected the narrative of the development of PPI in health research and increasing recognition of the value of patient knowledge over the past 15 years. However, this was tempered by Kat’s continuing frustrations with the narrowness of how PPI contributors are conceptualized, with researchers forgetting that “before we were patients we had careers.”

## Grace: “It’s My Professional Life Now”

### Personal Trajectory and Identity

The trajectory of Grace’s involvement with PPI covered a range of fields and involved multiple voluntary and paid roles over a couple of decades. She identified the starting point as taking up a paid role as coordinator of a mental health service user group, as a mental health service user herself. She described the activities they undertook, including advocacy and working with a local health authority to conduct research, and recognized that it was rather “unusual” for PPI at the time in the extent to which the research was “kind of co-produced.” From this “really positive experience,” Grace talked about becoming more involved in health research in an academic setting, as an employed “user-researcher,” and also supporting others to undertake PPI in their research.

Grace’s narrative portrayed a sense of “building up” a career, straddling PPI and academic research, alongside some voluntary roles. However, her account also featured multiple experiences of ill health and discrimination which disrupted her anticipated trajectory. She described developing a physical disability, managing “long term mental health problems,” and facing termination of her research contract in what she conveyed as a very unfair and upsetting process with a “long-lasting effect.” However, Grace reflected that her changing physical and mental health experiences had also given rise to new possibilities for involvement: “I am able to speak from quite a lot of different perspectives now.” She described a wide range of different PPI work that she has been involved in more recently, including being an advisor on several research networks and funding panels, and like other interviewees, she found it difficult to list all the different projects with which she is involved.

Grace’s narrative strongly positioned her PPI trajectory as occupying a professional status: “It’s my professional life now and it’s become more so as time has gone.” However, she also articulated the importance for her of continuing to align herself with her “grass-roots” personal health experiences, something which she is currently exploring through postgraduate study:

*I purposely continue doing, I don’t know, being a user on a reference group that kind of thing because I see that as kind of grass roots type work. . . . Yes and I purposely do that as well as doing other things like more strategic things, whatever because I just feel as though it keeps me grounded.*

She constructed an identity of her as a challenger, seeking to tackle injustices and unfairness relating to health and social care, but also in relation to PPI work. She conveyed frustrations with PPI structures, for example, how people are recruited into PPI roles rarely through open processes: “researchers have their pet patients and carers.” Grace also described personally facing barriers to some PPI work, due to a lack of accommodation of her physical mobility requirements, and described advocating for PPI coordinators and researchers to do more to ensure fair access to PPI roles. This identity of tackling “unfairness,” both for herself and for others, was something that Grace depicted as very important to her, but also “so exhausting.”

### Broader Narratives Represented

Narratives around money were prominent in Grace’s account, reflecting her blurred position as professional and volunteer PPI contributor, and her expectation for fairness and equality. She suggested the idea of her seeking financial remuneration as “kind of selfish . . . I have a mortgage to pay.” However, Grace also portrayed payment as an appropriate expression of the value of her, and others, contributions to PPI work, and of equality between PPI contributors and other professionals: “it’s not fair to take advantage of them [PPI contributors] and not pay them.” She also engaged with the narrative of PPI becoming increasingly participatory in moving toward “co-production,” but implicitly critiquing it through comparing her early PPI role with more recent experiences. She reflected on the “injustices” she has faced personally within PPI and research systems, and which she seeks to tackle “not only for my own personal gain but to try to improve things for other people.”

## Brendan: “Completely Changing the Landscape”

### Personal Trajectory and Identity

Brendan’s involvement in PPI started comparatively recently, and similar to Kendra and Kat, arose following a diagnosis, a degenerative condition with an unclear prognosis. Brendan described withdrawing from many aspects of his previous life immediately after the diagnosis, including taking early retirement: “I got the diagnosis and I was [told to] . . . put my affairs in order and basically go home and die.” After 6 months of “sitting indoors,” Brendan identified having a “light bulb moment” when attending a postdiagnosis support event, which motivated him to do as much as he could for as long as possible. This led to a trajectory of getting actively involved in health research “as a way of getting out of the house,” first as a participant and Brendan recounted participating in up to 20 studies in a year, and then as a PPI contributor. He highlighted an additional benefit of participating as having more access to his consultant clinician.

In Brendan’s narrative, the distinction between his involvement as a participant in research, and then in a PPI capacity, was not always clearly made. He depicted a rapidly expanding process of involvement across a range of activities; “there’s all sorts, I can’t remember them all.” Brendan described joining a research advisory group, drawing on his unfolding experiences of his condition to highlight issues that researchers had overlooked, which subsequently led to joining a steering group for research on one of these issues. He listed multiple other roles, focused particularly on pushing for resources for people with the same diagnosis, for example, working with a clinical commissioning group to “develop a pathway” for people in a similar situation to him. Amid this account of a busy PPI schedule, Brendan talked a little of his family life. Unlike Kat’s experience, he indicated that his wife was reluctant to engage with PPI, even though he had highlighted roles related to the experience of carers that would be relevant for her. Brendan considered that this might reflect the newness of his changed health status, and that his wife is still “dealing with [the] diagnosis,” so he tries not to “shove [PPI] in her face.” However, he also felt that his trips away for PPI work might offer his wife some welcome “respite” from their situation.

Within the PPI trajectory narrated by Brendan, he talked about the shift in the role he has taken in these opportunities, becoming increasingly “challenging”:

*So taking part in research is important for that reason, certainly a year ago it was getting me out of the house, getting me connecting with people. You know, and now I am moving more to challenging researchers when they don’t involve people with [condition], being involved in developing research.*

Through his narrative Brendan constructed an identity as an activist and advocate, frustrated with the services available to him and how his condition is little understood:

*You get professors challenging saying you can’t have [condition] because you are doing this, that and the other.*

Describing his role in pushing against the status quo for people sharing his condition also led Brendan to reflect on professional and family experiences. He stated that he had come to realize that his work now is a “continuation” of a role he had played within a historical employed position in health care, “challenging” poor care that he witnessed and “acting as an advocate for the people I was looking after.” He also recounted his father’s experience of being diagnosed with cancer with 2 weeks to live, but who had lived for a further 27 years and was actively involved in health care advocacy during that time. Against the uncertainty of his future, Brendan’s PPI narrative constructs a strong identity of activist.

### Broader Narratives Represented

Brendan engaged directly with narratives of activism and equality, drawing on the familiar “they say nothing about us without us” expression, for example, when describing a patient-led research initiative of which he was part. He conveyed an altruistic motivation in his work: “we’re doing it for those that can’t do it for themselves,” but with a stronger emphasis on justice and fighting for what people deserve than the familiar “giving back” PPI narrative. This also connected with Brendan’s account of the role of money in PPI work. Unlike Kendra and Bhai, he framed the financial aspects of PPI as a mode of challenging hierarchies and relations of power, rather than as income or mode of employment. He described deciding only to participate in events or workshops if expenses are paid—“if you don’t pay, I don’t do”—and had made this clear on his social media page. The financial narrative underpinned the construction of Brendan’s role in PPI of challenging inequalities around his condition.

## Discussion

We have presented narrative constructions of PPI in health research, to explore how these experiences intersect and are situated in relation to PPI contributors’ broader lives, rather than just in relation to the context of research. This work reflects the increasing imperative for PPI as part of the health research and funding infrastructure in the United Kingdom, and internationally. It also reflects the limited consideration in the literature to date of what meaning PPI holds for contributors beyond their identity within the research “system” or in relation to their health. Drawing on the narrative tradition in medical sociology for constructing and representing meaning around health, the body, and life (Hydén, 1997), we conducted repeat in-depth, narrative interviews with five experienced PPI contributors in the United Kingdom. While the narratives constructed by participants in the interviews were highly individualized and specific to their personal circumstances and experiences, there are broader issues implicated through these narratives that are relevant to understanding PPI as a social practice.

PPI literature and practice typically defines patients by disease/illness category or carer role, such as cancer, thus framing their “life-world authenticity” in terms of single identity construction that is of interest to the research agenda and knowledge production “system” (Bissell et al., 2018). However, our study highlights the numerous and shifting identities of PPI contributors that evolve over time and are “multiply constructed across different . . . discourses, practices and positions” (Hall, 1996, p. 4). While all participants identified a single health issue (their own, or that of a relative) as the starting point for their PPI journeys, they narrated many different and overlapping positionalities around their PPI experiences. These included changing employment and professional status; identifying and pursuing opportunities for activism and advocacy; evolving caring, family, and social relationships; shifting financial status; and changing health status.

As such, attempting to understand PPI contributors’ experiences from the starting point of a particular disease or caring role, risks privileging PPI’s meaning only in terms of the kinds of experience that health researchers are most interested in (Thompson et al., 2012). Instead, our research highlights the intersection of health, employment, financial, social, and political identities, and how they may shift over time, as fundamental to understanding the value and experiences of PPI in contributors’ own “life-worlds.” This adds to debates challenging the representative role that contributors are often (implicitly) asked to play through PPI (Maguire & Britten, 2018), by dismantling the assumption that it is meaningful to attribute single health or care-related identities to contributors.

Furthermore, the act of narrating PPI journeys itself contributed to the construction of participants’ identities (Hydén, 1997). This provided a mechanism through which participants could reflect on and position themselves at different points in time, such as Brendan’s realization of his PPI work as an extension of the advocate role he adopted in previous employment. Our participants demonstrate how new forms of meaning and self-perception can arise through the intersection of PPI and other areas of their lives, over time. Some literature has identified the potential for involvement in PPI to enable reconfiguring of the self along “more positive or constructive lines” (Thompson et al., 2014, p. 51), particularly when a serious health event has “disrupted” contributors’ lives. Certainly, there are echoes of this perspective in our findings, for example, Kendra reflecting on PPI enabling a positive renegotiating of her status in relation to professional work. However, importantly, our research highlights how this reflection and repositioning may not always be positive, as implied in much PPI literature. For some, like Bhai, Grace, and Brendan, these experiences may bring into sharp relief uncomfortable positions of inequality, injustice, and frustration, faced by past and current selves, and potential future selves. This evokes the reflective and “awakening” potential of participation (Campbell, 2005): through PPI, contributors construct new interpretations of their positions within the world, and of the benefits *and* limitations of these positions.

Our participants’ narratives also show that the very spaces and processes of PPI (such as bureaucratic university payment systems) may be experienced by some as exclusionary and difficult to navigate, further emphasizing their difference and disconnection from those who are more established in these spaces, such as professional researchers. The tendency in the literature to frame PPI experiences as largely positive and productive (although with some challenges along the way) likely reflects the imperative for public participation now embedded within the health research system. The potential for PPI processes to exclude minority groups (including ethnic minorities) and those with the least capacity to participate has been acknowledged (Dawson et al., 2018; Schwartz et al., 2019). Yet, little attention has been paid to how the experiences of those actively involved in PPI may reflect and even reinforce broader social relations of inequality and exclusion beyond the research context. Our research indicates this is an important area for further consideration and emphasizes the value of narrative approaches for exploring identity in PPI by revealing disjunctures between anticipated, or hoped-for, and experienced realities (Vindrola-Padros & Johnson, 2014).

By exploring PPI from the framing of contributors’ own “life-worlds,” we can begin to understand how its value and meaning for contributors are intertwined with wider systems of social, political, and economic power, within which the “ivory tower” of health research (Locock et al., 2017) is also situated. As indicated in Kat, Grace, and Brendan’s narratives, involvement in PPI over time may increase confidence to challenge research structures and provide opportunities for activism and advocacy in relation to health and the production of knowledge. Yet, while these motivations echo trends toward “co-production” in research (Hughes & Duffy, 2018), our participants’ experiences also revealed ongoing frustrations they face in terms of the impact of their involvement, struggling against embedded hierarchies of power. Understanding these experiences in PPI and how they sit within contributors’ broader lives should help researchers and those coordinating PPI to better support the different motivations and needs of contributors, and reflect more critically on the power relations embedded in health research.

Participants’ varied narratives also demonstrated the framing of PPI as meaningful “work.” This corresponds with existing literature highlighting contributors’ accounts of developing greater agency through PPI experiences, particularly following the end of a professional career (Thompson et al., 2014), and with literature on volunteering as forms of purposeful and productive work (Kelemen et al., 2017). Ongoing debates around the “professionalization” of PPI contributors as they build experience in research over time have explored some aspects of PPI as productive work in terms of the status and skills established as contributors become more “expert” (Thompson et al., 2012). Our findings extend this knowledge, illustrating additional forms of value that PPI as “work” may have for contributors, as a substitution for, or type of formal, paid employment (Bhai and Grace), and as a mechanism for instigating change and having impact on the world (Brendan and Grace). Importantly, these forms of value must be recognized for their meaning and application beyond the health research context, revealing the broader significance of PPI work as a purposeful way of “being in the world” (Yeoman, 2014). As such, our findings reflect some of the critiques of the “professionalization” narrative, which is built on the simplistic (research-centered) dichotomy of the “naïve” lay person and the “expert” research professional (Martin, 2008). Our research highlights the necessity of acknowledging the range of knowledges, meanings, and value that PPI-as-work can develop, to recognize more fully what PPI contributors bring into the research context, in addition to health and/or caring experience.

Furthermore, our research highlights that we must also pay more attention to the financial dimension of PPI as meaningful “work,” not only in terms of a symbolic “recognition of their contributions to the research process” (see INVOLVE, 2020), but also in terms of how PPI work makes possible and constrains other dimensions of life. Literature on PPI and volunteering tends to focus on altruistic and reciprocal relations as key motivations for involvement (see, for example, Thompson et al., 2014). However, in our research, the narratives of Kendra, Bhai, and Grace indicate that money received for doing PPI was also an important way for them to be in the world, in terms of offering a basic income. This perspective could be interpreted in relation to the “professionalization” of PPI debates (Locock et al., 2017), whereby giving financial recompense for both the “lay” and “professional” contributions (albeit on considerably different scales) may further blur the distinction between the two.

Yet, our findings show how the financial side of PPI also acts as a strong reminder for some contributors of their positions of relative inequality and constraint, largely excluded from the formal employment and/or income they would like to have. In doing so, it also raises felt tensions for contributors between the expected altruistic motivation for doing PPI, and the desire (even necessity) within the context of their life-world to be paid for the work. Finally, expectations for financial recompense for PPI work may be mobilized as a form of activism or mechanism of “collective empowerment” (Potter, 2015). For Grace and Brendan, questioning payment conventions served to highlight and challenge the unequal power structures surrounding the production of research knowledge. Thus, our research indicates that the financial dimensions of PPI need deeper exploration to unpack these varied sets of relations, and the role that money plays in mediating the power, status, and capabilities of contributors both within and beyond the research context.

### Limitations

Our research design included a deliberately small sample of five participants, to facilitate depth of understanding of their individual narratives and experiences, but which has limited our capacity to produce more generalized accounts that are directly transferable across areas of PPI or in countries other than the United Kingdom. However, we believe our research has identified important aspects of contributors’ experiences which highlight the complex intersection between broader social, financial, health, and employment experiences and meaning constructed around PPI. Further research is recommended for exploring these aspects across a broader sample of participants, to understand more deeply how individual positions intersect PPI experiences, including across demographic categories such as gender, ethnicity, and socioeconomic status. This is particularly important given acknowledged barriers to involvement in PPI among minority groups (Dawson et al., 2018).

While our repeat narrative interviews enabled considerable depth of understanding of how PPI contributors discursively construct their identities and experiences, we recognize that this method privileges a certain type of communication: extended talk and storytelling (Hurdley, 2006). Due to the varied length of interviews in this study, it is possible that some participants were more comfortable with the extended narration of their experiences than others. Future research would benefit from involving varied approaches for facilitating narratives, such as visual methods. Also, while our research question focused on PPI in health research, participants’ narratives did not stick only to this category of involvement, and it was not always possible to determine which kinds of PPI activity participants were reflecting on. This indicates that it is not necessarily meaningful to set health research apart from their other participatory activities, such as involvement in health and social care services, and education. Further work is needed around how PPI for health research is experienced within and across broader participatory spaces.

## Conclusion

Amid the increasing expectation for PPI in health research in the United Kingdom and beyond, careful consideration of the multiple identities and experiences of PPI contributors is required. Our research shows the importance of understanding PPI as a social practice that shapes and is shaped by contributors’ evolving relationships with family and friends, work and financial status, health and well-being, and positions within societal structures of exclusion, inequality, and injustice. Those organizing and advocating for PPI need to avoid categorizing contributors only by a single health or caring-related identity and work harder to engage the dynamic and evolving knowledges that PPI contributors can usefully bring to the health research context. The health research system also needs to establish structures that better support the diversity of experiences of PPI contributors, acknowledging that their contributions to research are connected to the broader social, political, and financial (as well as health) positions they find themselves in. Without taking seriously the range of dynamic experiences, meanings, and motivations characterized and produced through PPI contributors’ complex narratives, we risk, as Madden and Speed (2017, p. 5) argue, “experts by experience . . . being reduced to another commodity.”
